# Draft Genome Sequence of *Bacillus cytotoxicus* CVUAS 2833, a Very Close Relative to Type Strain NVH 391-98 Isolated from a Different Location

**DOI:** 10.1128/genomeA.00901-15

**Published:** 2015-08-20

**Authors:** Maria-Elisabeth Böhm, Christopher Huptas, Viktoria Magdalena Krey, Siegfried Scherer

**Affiliations:** Lehrstuhl für Mikrobielle Ökologie, Zentralinstitut für Ernährungs- und Lebensmittelforschung (ZIEL), Technische Universität München, Freising, Germany

## Abstract

We report the draft genome sequence of *Bacillus cytotoxicus* CVUAS 2833, isolated from potato puree in Germany (2007), which is—despite its clearly different source—very similar to the type strain *B. cytotoxicus* NVH 391-98 isolated in France (average nucleotide identity, 99.5%).

## GENOME ANNOUNCEMENT

The highly enterotoxic *Bacillus cereus* NVH 391-98 was discovered in vegetable puree, which caused severe food poisoning including fatalities (France, 1998) (1). In this strain, a new cytotoxin CytK-1 was identified. Subsequently, the distantly related homolog CytK-2 was discovered in other strains of the *B. cereus* group which display considerably attenuated toxicity (2). *B. cereus* NVH 391-98 was published in 2013 as the type strain of the new species *Bacillus cytotoxicus* due to the presence of the *cytK-1* gene, its thermotolerance (growth at up to 50°C), a distinctive fatty acid profile, DNA-DNA hybridization, and multilocus sequence typing (MLST) (3). *B. cytotoxicus* CVUAS 2833, which is one of only five known *B. cyototoxicus* strains, was isolated spatially and chronologically separate, but affiliated to *B. cytotoxicus* by 16S rRNA gene sequence and MLST comparison (3, 4). To date (June 2015), only the genome sequence of the type strain has been available.

We sequenced *B. cytotoxicus* CVUAS 2833 on the Illumina MiSeq platform and obtained a library of 3,185,212 reads. About 72.2% of the reads passed quality filtering (length, ≥80%; Phred score, *Q* ≥ 30) and were assembled to 36 contigs using ABySS version 1.3.7 with an assembly size of 4,127,075 bp and a G+C content of 35.74%. The assembled contigs were submitted to GenBank and annotated by NCBI’s prokaryotic genome annotation pipeline (PGAP). BLAST analysis revealed the presence of the distinct *B. cytotoxicus* variant of the enterotoxin operon *nhe* (5) in strain CVUAS 2833 with only a single nucleotide difference to NVH 391-98 *nhe*. *B. cytotoxicus* CVUAS 2833 does not contain the *ces* cluster encoding the emetic toxin or the *hbl* enterotoxin genes. Whole-genome pairwise average nucleotide identity (ANI) of 99.5% supports the exceptionally high similarity to *B. cytotoxicus* NVH 391-98. Additionally, Gegenees 2.2.1 was used to compare whole-genome similarity in a fragmented alignment (BLAST 2.2.29+) at a fragment size of 200 bp and a comparison step size of 100 bp (6). In contrast to ANI, this approach applies stricter settings (7) and does not exclude dissimilar sequences (8). *B. cytotoxicus* NVH 391-98 and CVUAS 2833 showed 93.5% identity in the fragmented all-all comparison.

These genomic and genetic similarities confirm a clonal character of the *B. cytotoxicus* lineage within *B. cereus sensu lato*. All known *B. cytotoxicus* strains originate from different food sources (1, 9), but are similar on a MLST basis (3). The only well-analyzed clonal phylogenetic cluster in *B. cereus sensu lato* is *Bacillus anthracis*. The *B. anthracis* linage is evolutionarily young and demarcation of *B. anthracis* at a species level is quite debatable (10, 11). In contrast, *B. cytotoxicus* seems to be a much older and clearly discernible branch within *B. cereus sensu lato* (12).

### Nucleotide sequence accession numbers.

The draft genome sequence of *B. cytotoxicus* CVUAS 2833 has been deposited at DDBJ/EMBL/GenBank under the accession no. JYPG00000000. The version described in this paper is version JYPG01000000.
